# Impact of Detailed Versus Generic Instructions on Fine-Tuned Language Models for Patient Discharge Instructions Generation: Comparative Statistical Analysis

**DOI:** 10.2196/80917

**Published:** 2025-10-30

**Authors:** Muneerah Alqahtani, Abdullah Al-Barakati, Fahd Alotaibi, Mohammed Al Shibli, Saad Almousa

**Affiliations:** 1 Information System Department Faculty of Computing and Information Technology King Abdulaziz University Jeddah Saudi Arabia; 2 Information Systems Department King Khalid University Abha Saudi Arabia; 3 Primary Health Care Center Aseer Health Cluster Abha Saudi Arabia; 4 College of Medicine King Khalid University Abha Saudi Arabia

**Keywords:** discharge instructions, large language models, clinical text generation, instruction tuning, medical natural language processing, open-source large language models, patient safety, prompt engineering

## Abstract

**Background:**

Discharge instructions are essential for patients after hospital care but are time-consuming to write. With the rise of large language models (LLMs), there is a strong potential to automate this process. This study explores the use of open-source LLMs for generating discharge instructions.

**Objective:**

We investigated whether a Mistral model can reliably generate patient-oriented discharge instructions. Two distinct instruction-tuning paradigms were compared, each using a different mechanism for embedding guidance during fine-tuning.

**Methods:**

In our experiment, we applied Mistral-NeMo-Instruct, an LLM, in combination with 2 distinct instruction strategies for fine-tuning. The first were detailed instructions tailored to the task of discharge instruction generation. The second was a basic instruction with minimal guidance and no task-specific detail. The independent variable in this study is the instruction strategy (detailed vs generic), while the dependent variables are the evaluation scores of the generated discharge instructions. The generated discharge instructions were evaluated against 3621 ground-truth references. We used Bilingual Evaluation Understudy (BLEU-1) to BLEU-4, Recall-Oriented Understudy for Gisting Evaluation (ROUGE-1, ROUGE-2, and Recall-Oriented Understudy for Gisting Evaluation—Longest Common Subsequence), SentenceTransformer similarity, and Bidirectional Encoder Representations From Transformers Score as evaluation metrics to assess the quality of the generated outputs in comparison to the corresponding ground-truth instructions for the same discharge summaries.

**Results:**

The detailed instruction model demonstrated superior performance across all automated evaluation metrics compared with the generic instruction model. Bidirectional Encoder Representations From Transformers Score increased from 78.92% to 87.05%, while structural alignment measured by Recall-Oriented Understudy for Gisting Evaluation—Longest Common Subsequence improved from 8.59% to 26.52%. N-gram precision (BLEU-4) increased from 0.81% to 21.24%, and the Metric for Evaluation of Translation With Explicit Ordering scores rose from 15.33% to 18.47%. Additional metrics showed consistent gains: ROUGE-1 improved from 16.59% to 42.72%, and ROUGE-2 increased from 1.97% to 45.84%. All improvements were statistically significant (*P*<.001), indicating that detailed, task-specific instruction design substantially enhances model performance.

**Conclusions:**

The use of detailed, task-specific instruction strategies significantly enhances the effectiveness of open-source LLMs in generating discharge instructions. These findings indicate that carefully designed instructions during fine-tuning substantially improve model performance.

## Introduction

### Background

The quality of writing discharge instructions represents a challenge in health care hospitals. Discharge instructions serve as essential guides for posthospitalization care, but their crafting needs significant physician time within already demanding clinical workflows. Studies show that spending too much time on documentation is directly linked to physician burnout and poor work-life balance. Some providers spend over 6 hours each week on after-hours documentation [1,2]. Physicians showed low satisfaction with their electronic health records (EHRs) and computerized order systems (Computerized Physician Order Entry) because of the large amount of time spent on clerical work to write patient records. This growing burden highlights the urgent need for supportive tools to automate the process [3].

### Current Solutions and Their Limitations

The use of predefined templates within EHRs is a widespread strategy for producing discharge instructions. However, these existing solutions have their own limitations. While they are intended to improve efficiency and ensure key information is included, they often fall short in practice. Evidence shows that even when disease-specific templates are available, they are frequently underused and fail to enhance the readability of patient instructions [4]. As a result, each discharge requires manual customization, where doctors frequently modify or rewrite discharge instructions to reflect individualized care plans. They add personalized medications, tailor activity restrictions, and simplify medical terminology for patient understanding [5,6]. This time-consuming process leads to inconsistent quality between providers. Under discharge time pressure, important information may be missing or written in ways patients cannot understand [6].

### Language Models: A Promising Solution

According to a recent scoping review by Meng et al [7], large language models (LLMs) have been actively explored in several areas of medicine, including clinical documentation support. The review, which analyzed over 550 studies, found that LLMs have the potential to reduce clinician workload and streamline routine writing tasks. By leveraging their ability to understand and generate medically relevant content, LLMs present a promising solution to address the time burden associated with clinical documentation [7].

### Optimizing LLMs Through Instruction Design for Discharge Instructions

While LLMs show a great promising solution to generate discharge instructions, their effectiveness depends largely on how they are guided, especially through prompt design.

Two primary optimization approaches have emerged: (1) prompt engineering at inference time, where users modify input prompts without changing the model [8]; and (2) instruction tuning during training, where models are fine-tuned with task-specific instructions [9].

While prompt engineering has shown significant effects on LLM performance in general domains [8,10]. Instruction tuning offers deeper adaptation by incorporating domain knowledge directly into model parameters. In medical contexts, studies have demonstrated that carefully designed prompts can improve diagnostic reasoning and clinical documentation quality [8,11].

### Aim of This Study

While LLMs are gaining popularity in health care, it remains unclear how much their performance depends on the way they are guided, especially in the context of generating discharge instructions. To investigate this, we conducted a comparative study evaluating 2 instruction strategies. We used 2 instruction strategies for generating discharge instructions. The tailored method used detailed instructions specifying medical roles (eg, doctors or physicians) and clear guidelines tailored to hospital-specific needs. The generic method used short, open-ended instructions. Both models were trained on the same set of real discharge summaries from the Medical Information Mart for Intensive Care IV (MIMIC-IV) database [12] and evaluated using standard metrics, including Bilingual Evaluation Understudy (BLEU) [13], Recall-Oriented Understudy for Gisting Evaluation (ROUGE) [14], Metric for Evaluation of Translation With Explicit Ordering (METEOR) [15], and Bidirectional Encoder Representations From Transformers Score (BERTScore) [16], to assess performance.

The primary objective was to assess whether more structured instructions lead to more accurate, relevant, and patient-friendly discharge instructions. This study aimed to determine if structured instructions generate accurate, relevant, and patient-friendly discharge instructions. More broadly, the study explores providing practical insights for implementing LLMs in hospital settings.

The integration of LLMs into hospitals’ workflows is expected to offer a solution to reduce the documentation burden. LLMs could help physicians focus more on patient care.

## Methods

### Ethical Considerations

This study used the MIMIC-IV database [12], a publicly available dataset containing deidentified health information from critical care patients. The database was approved by the institutional review board of the Massachusetts Institute of Technology (IRB #2001P001699) and was granted a waiver of informed consent in accordance with the Health Insurance Portability and Accountability Act (HIPAA) Safe Harbor provisions. All authors completed the Collaborative Institutional Training Initiative “Data or Specimens Only Research” training and signed the PhysioNet Credentialed Health Data Use Agreement. The lead author (MA, credential approved on July 8, 2024) maintained an active credential for database access. To protect participant privacy, all data were used strictly in compliance with the data use agreement. No attempts were made to reidentify individuals, and all analyses were performed on secure, access-controlled systems. Because the dataset is fully deidentified and publicly available, no participant contact or compensation was involved.

### Study Design

**Figure 1:**
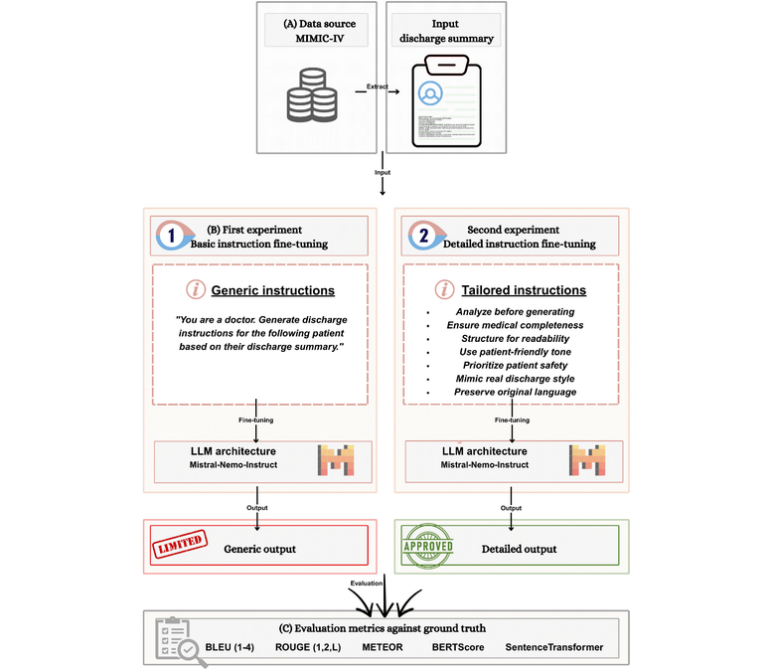
Study workflow. (A) Data preparation (35,851 pairs from MIMIC-IV). (B) Distinct fine-tuning on Mistral-NeMo-Instruct with generic versus tailored instructions. (C) Evaluation with BLEU, ROUGE, METEOR, BERTScore, and SentenceTransformer similarity. BERTScore: Bidirectional Encoder Representations From Transformers Score; BLEU: Bilingual Evaluation Understudy; LLM: large language model; METEOR: Metric for Evaluation of Translation With Explicit Ordering; MIMIC-IV: Medical Information Mart for Intensive Care IV; ROUGE: Recall-Oriented Understudy for Gisting Evaluation.

This was a comparative study based on an experiment of testing 2 instruction strategies for generating discharge instructions: generic instructions (21 words) versus tailored, detailed instructions (587 words). We tested these strategies by fine-tuning the same base model (Mistral-NeMo-Instruct) twice. A schematic of the workflow is shown in Figure 1.

The experiment had 2 sides, one with generic instructions and the other with tailored instructions. During inference, each model used its corresponding instruction type when generating discharge instructions. For example, the model trained with generic instructions received the same 21-word generic prompt at inference time, while the model trained with tailored instructions received the same 587-word detailed prompt. This approach allowed us to evaluate the full impact of instruction design on model performance throughout both training and inference phases.

Our experiment followed 3 steps. First, we prepared the data by extracting 35,851 discharge summary-instruction pairs from MIMIC-IV. Second, we fine-tuned 2 models using the Mistral-NeMo-Instruct architecture. One was fine-tuned with generic instructions, and one with tailored instructions. Third, we evaluated the generated discharge instructions using multiple metrics (BLEU, ROUGE, METEOR, BERTScore, and SentenceTransformer similarity). This design lets us measure how much tailored instructions improved discharge instruction generation compared to generic ones.

### Data

#### Data Source and Description

This study used the MIMIC-IV database (version 2.2, released July 23, 2024), a publicly available collection of 364,627 deidentified health records from patients admitted to intensive care units or emergency departments between 2008 and 2022. Managed by Massachusetts Institute of Technology researchers, MIMIC-IV was a widely respected resource in medical research due to its continuous maintenance and evidence-based clinical documentation.

We built our data from the discharge.csv file, using notes labeled as “Discharge summary.” These summaries included comprehensive patient details such as symptoms, patient history, medications before and after admission, laboratory results, and most importantly, the discharge instruction section. In our experiment, each summary served as input for our model, while the discharge instruction sections extracted from these summaries were used as expected output for model training. We used discharge instruction sections as the ground truth in the evaluation phase.

We chose MIMIC-IV for its reliability because patients’ records were real and written by health care professionals. Additionally, the dataset contained diverse, real-world patient cases. It was a widely respected resource in medical research due to its continuous maintenance and evidence-based clinical documentation [12].

#### Preprocessing and Formatting

Our preprocessing pipeline extracted full discharge summary text from the report column of the MIMIC-IV database [12]. We constructed input or output pairs by separating the whole discharge summary documentation (input) and the “Discharge Instructions” section (output) using regular expressions Regex [17].

We applied some filtering steps. We excluded records of patients with discharge conditions labeled as “Expired,” “Deceased,” or “Gone Away,” retaining only those of living patients, who were eligible for discharge instructions. We also excluded records of patients who were transferred to other medical facilities such as another hospital, because their discharge instructions would be issued by the facility that received them. Additionally, we excluded summaries that exceeded 20,000 characters to reduce the noise from outliers. We dropped all records with instructions that had fewer than 5 words because they may not have carried meaningful information for the patient.

**Figure 2:**
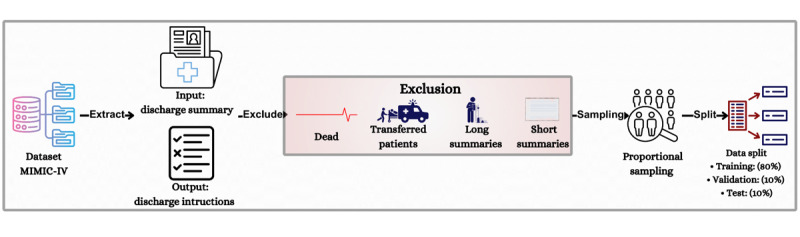
High-level overview of the preprocessing pipeline. MIMIC-IV: Medical Information Mart for Intensive Care IV.

Given the large size of the original dataset (364,627 records), we performed proportional sampling to create a smaller but representative dataset. We ensured that the dataset still reflected the original distribution of medical services. Analysis of the service distribution revealed that the department of medicine dominated with 195,533 cases (59.1% of total admissions). Our proportional sampling reduced the dataset from 364,627 records to 35,851 records while preserving the relative distribution across medical services. Therefore, the final dataset contained 35,851 records, as illustrated in Figure 2, which provides a high-level overview of the preprocessing pipeline.

For instruction fine-tuning, we used predefined templates based on the Mistral-NeMo-Instruct format. The template comprised 4 core components: role (asking the model to act as a health care provider), instructions (guidelines for structuring output), input (the discharge summary), and output (the discharge instructions). To assess how the complexity of instructions affected the performance of the model when asked to generate discharge instructions, we developed 2 template variants: a simple, generic version and a detailed, task-specific version. The detailed template incorporated advanced prompting techniques, including acting as a physician, analyzing discharge summaries to identify medical details. The detailed template also stressed using simple, easy-to-understand instruction that is friendly for patients.

#### Final Dataset

Our final dataset comprised 35,851 high-quality discharge summary-instruction pairs. The dataset was split into training (n=28,673, 80%), validation (n=3557, 9.9%), and test sets (n=3621, 10.1%). Table 1 summarizes the number and percentage of samples in each split.

**Table 1:** Dataset splits for model development and evaluation.

Dataset	Samples, n (%)
Training	28,673 (80)
Validation	3557 (9.9)
Test	3621 (10.1)
Total	35,851 (100)

Statistical analysis of token counts revealed important characteristics of our data. Discharge summaries ranged from 276 to 3066 tokens with a mean of 1437 (SD 550; median 1414, IQR 1012-1876) tokens, while discharge instructions were considerably shorter, ranging from 68 to 420 tokens, with a mean of 176 (SD 72; median 157, IQR 118-214) tokens. The 90th percentile was 2193 tokens for discharge summaries and 291 tokens for discharge instructions, indicating that most documents fit comfortably within our model’s context window, as detailed in Table 2.

**Table 2:** Token distribution statistics for discharge summaries and instructions.

Statistic	Discharge summaries	Discharge instructions
Mean (SD)	1437 (550)	176 (72)
Range	276-3066	68-420
Median (IQR)	1414 (1023-1812)	157 (118-223)
90th percentile	2193	291

### Instructions Design

#### Overview

**Figure 3:**
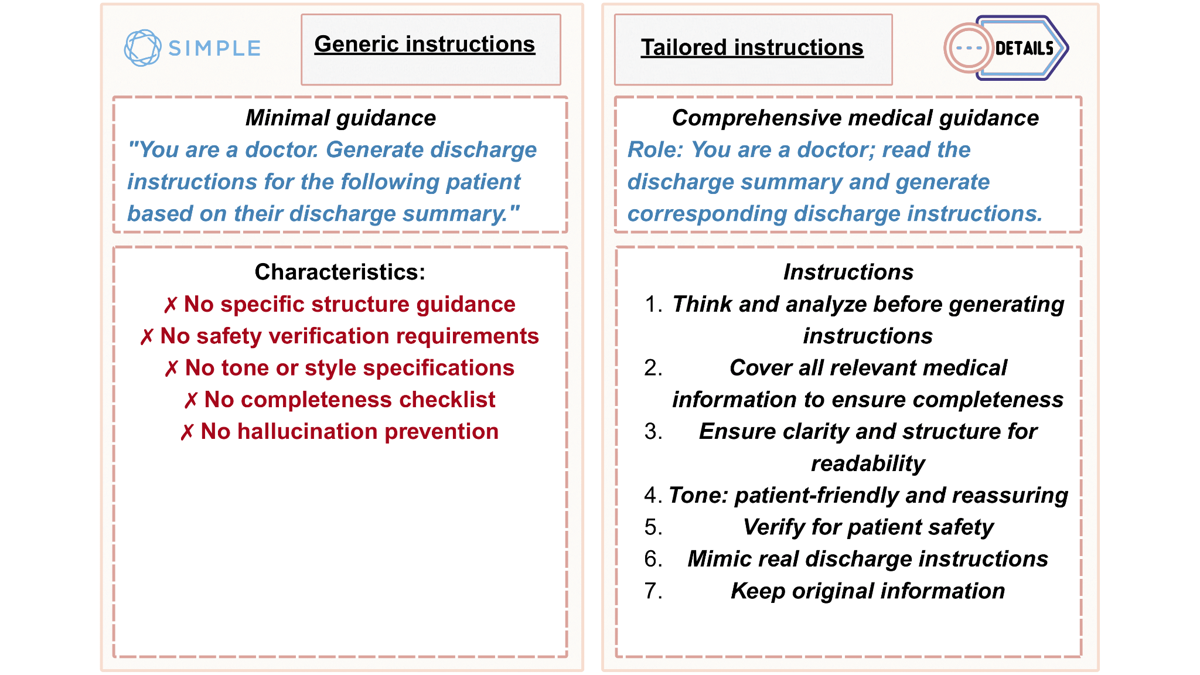
Generic versus tailored instruction design used during training and inference. The generic prompt (21 words) provides minimal task guidance. The tailored prompt (587 words) encodes structure, tone, safety checks, and completeness criteria.

The core innovation of this study lay in comparing 2 distinct prompting strategies for instruction fine-tuning: a generic minimal instruction versus a tailored comprehensive instruction. Both instructions were used during model training and inference. This section details the design rationale and specific content of each instruction (Figure 3).

#### Generic Instructions

The generic instructions were designed to represent a minimal instruction approach. It provided only an essential task definition without domain-specific guidance. For example, “You are a doctor. Generate discharge instructions for the following patient based on their discharge summary.”

These 21-word instructions were deliberately designed to test whether LLMs could generate adequate discharge instructions with minimal guidance. It provided only 3 key elements: role identification (“You are a doctor”), task specification (“Generate discharge instructions”), and structural markers for input and output. We did not add additional constraints or formatting requirements.

#### Tailored Instructions

The tailored instructions were designed to incorporate essential domain-specific guidance based on analysis of the final dataset. We recognized that discharge instructions lacked universal standardization across health care institutions. Thus, these 587-word instructions were designed to capture common features and maintain diverse documentation styles.

The instruction text was derived from MIMIC-IV [12] discharge instructions using manual observation rather than rule-based extraction. A qualitative review was conducted to identify recurring elements and their typical order. The instructions were drafted from these observations. Then, we ran pilot trials on 100 summaries, monitored metric behavior, and made minor edits to the instructions. The final instruction text was fixed before fine-tuning and final evaluation.

Our analysis of discharge instructions revealed regular patterns that helped formulate the tailored instructions. These instructions often started with a greeting, such as “Dear [Patient],” followed by the reasons behind admission, including presenting symptoms, a summary of treatments provided, and a plan for ongoing care. Additionally, physicians consistently used clear, simple, and patient-friendly language to ensure readability.

We confirmed that most instructions included essential components such as (1) reason for admission, whether diagnosis or presenting symptoms; (2) key treatments and procedures performed during hospitalization; (3) medication changes with clear dosing instructions; (4) follow-up appointments and monitoring requirements; and (5) any warning signs that needed immediate medical attention.

Both instructions were integrated into the Mistral-NeMo-Instruct instruction template format during training, which included four core components: (1) the role, (2) instructions, (3) input, and (4) output. Each discharge summary was paired with its corresponding instructions and target output, allowing the model to learn distinct generation patterns. The full 21-word generic prompt and the complete 587-word tailored prompt are provided in Multimedia Appendix 1.

### Model

#### Model Selection Criteria

We scanned existing models in order to select an efficient base model for discharge instruction generation. We considered the following factors: (1) demonstrated performance on medical and instructional tasks, (2) computational efficiency enabling training on available hardware, (3) support for extended context windows to accommodate lengthy discharge summaries, and (4) open open-source model. Based on these criteria, Mistral-NeMo-Instruct was selected as our foundation model.

#### Mistral NeMo Instruct

Mistral NeMo 12B, developed by Mistral AI in collaboration with NVIDIA and released in March 2024, represented a significant advancement in efficient LLM design. This 12-billion parameter model introduced enhanced reasoning abilities while maintaining computational efficiency, which made it accessible to researchers. A key feature was its expanded context window supporting 128,000 tokens, allowing it to process even the longest discharge summaries in our dataset without truncation concerns. An important practical consideration was that the entire 12-billion parameter model could run on a single high-end consumer graphics processing unit (GPU).

#### Model Configuration

We used Unsloth training speedup, parameter-efficient fine-tuning for low-rank adaptation implementation and bitsandbytes for 4-bit quantization. This stack enabled efficient training on a single GPU while maintaining model performance.

### Training

#### Hardware and Environment

Model training and evaluation were conducted on Vast.ai using an NVIDIA H200 GPU with 140 GB of video random access memory. The machine environment included 192 central processing unit cores, 258 GB RAM, and CUDA (version 12.6). Training was implemented using the Unsloth framework built on top of Transformers, TRL, and Safetensors libraries. All experiments were executed in a Linux environment with Python (version 3.10; Python Software Foundation).

#### Training Configuration

We fine-tuned the base model unsloth/Mistral-Nemo-Instruct-2407-bnb-4bit using low-rank adaptation with the configurations presented in Table 3.

**Table 3:** Training configuration.

Setting	Values
Training samples	28,673
Validation samples	3557
Epochs	7 (with different convergence rates for basic and detailed models)
Batch size	1
Learning rate	4×10^–5^
Maximum sequence length	15,000 tokens
Context window optimization	128,000
Instructions fine-tuning format	Mistral-NeMo-Instruct style (21-word generic or 587-word detailed instruction)
Few-shot examples	We used only 2 examples during inference time

### Evaluation

#### Overview

To evaluate models, we used multiple automated metrics to assess the quality of generated discharge instructions. The evaluation was applied to 3621 records, which represented 10% of the dataset. All metrics compared model-generated instructions against the ground truth, physician-written reference instructions.

#### Evaluation Metrics

We selected evaluation metrics that captured different aspects of discharge instruction quality. Surface-level similarity was assessed using BLEU [10] scores (BLEU-1 through BLEU-4), measuring n-gram precision at increasing granularity levels, and ROUGE scores [11] (ROUGE-1, ROUGE-2, and Recall-Oriented Understudy for Gisting Evaluation—Longest Common Subsequence [ROUGE-L]) evaluated both unigram or bigram overlap and longest common subsequences. Additionally, METEOR scores [12] provided a synonym-aware evaluation particularly valuable for medical terminology variations where “hypertension” and “high blood pressure” conveyed identical meaning.

Semantic similarity was measured using the official BERTScore [13] implementation (bert-score library), which computed *F*_1_-scores based on contextualized embeddings from roberta large model, providing a more nuanced assessment than simple word overlap, and SentenceTransformer [18] similarity using all-MiniLM-L6-v2 to generate dense vector representations and calculate cosine similarity.

#### Cross-Model Validations

The external validation was conducted with 200 cases to verify that the benefit of detailed task instructions is not specific to one architecture. We used 4 additional models from different LLM families. We compared basic versus detailed prompting (without fine-tuning) for GPT-4 [19], GPT-3.5-turbo [20], Mistral-7B-Instruct [21], Qwen2.5-3B [22], and Llama-3.2-3B [23]. Preprocessing, prompts, and scoring followed the same protocol as the main study.

#### Statistical Analysis

The Wilcoxon signed rank [24] statistical test was used because our evaluation involved paired, nonparametric text data and did not assume a normal distribution. This test allowed us to effectively compare discharge instructions generated by 2 different models on the same sample of 3621 discharge summaries. Additionally, we calculated the rank-biserial correlation, which is necessary for computing the effect size. This metric helps quantify the practical significance of the observed differences. All analyses were performed using Python’s SciPy library, evaluating performance metrics (BLEU [10], ROUGE [11], METEOR [12], and BERTScore [13,15]) across the full evaluation dataset.

#### Qualitative Analysis

A targeted, case-based qualitative review was conducted. We first identified candidate instances using quantitative signals (eg, low ROUGE-L). From these samples, cases that reflected safety-relevant errors were purposively sampled. The illustrative cases were reported to contextualize model behavior. We did not estimate prevalence or conduct formal frequency estimates because of the difficulty of reliably labeling possible generation errors. Although various failure modes occurred in the discharge instructions generated by both models, our analysis was focused on 2 particularly concerning error types (omission and hallucination) because of their potential impact on patient safety and continuity of care. We defined omission as the absence of clinically salient information in the generated discharge instructions that was present in the reference text and was necessary for safe and effective patient care. We also defined hallucination as the introduction of clinical information in the generated discharge instructions that was not present in the reference text and was not entailed by it, especially when such additions could affect patient care. We presented discharge-instruction cases, where the basic and detailed models produced outputs demonstrating these errors.

#### Human Evaluation

Two consultant physicians evaluated 40 paired discharge instructions, with each assessing 20 cases within their specialty expertise. A plastic surgeon with microsurgery subspecialty evaluated surgery and neurology cases, while a family medicine specialist evaluated medicine and orthopedics cases. Cases were drawn from 4 services (medicine, neurology, surgery, and orthopedics), 10 samples from each. Safety was rated in 3 categories: safe, safe but needs minor changes, and not safe at all. Ratings required explicit red-flag instructions. Outputs with clearly unsafe statements (eg, dose doubling, mixing alcohol with opioids or sedatives, advice to ignore worsening symptoms, or unrestricted immediate activity) were classified as not safe at all. For each case, physicians received the deidentified discharge summary and both model outputs and recorded their ratings. Analyses used descriptive counts and proportions only. Raters were blinded to model identity; both followed a predefined rubric (Multimedia Appendix 2). We did not compute interrater agreement due to split-by-specialty assignment.

## Results

### Model Performance

We evaluated the performance of 2 instruction-tuned models: the generic and tailored models. Table 4 presents the comprehensive evaluation results comparing the generic and tailored models across all metrics on the test set of 3621 discharge instructions.

**Table 4:** Comparison of automatic evaluation metrics for discharge instruction generation using basic versus detailed fine-tuning strategies^a^.

Metric	Generic instructions+fine-tuning	Detailed instructions+fine-tuning^b^
METEOR^c^	15.33	18.47
BERTScore^d^	78.92	87.05
ROUGE-1^e^	16.59	42.72
ROUGE-2	1.97	45.84
ROUGE-L^f^	8.59	26.52
BLEU-1^g^	13.63	32.86
BLEU-2	4.46	33.35
BLEU-3	1.76	25.13
BLEU-4	0.81	21.24
SentenceTransformer similarity	11.91	74.90

^a^The detailed+fine-tuning model consistently outperforms the generic+fine-tuning model across all metrics, with notable improvement.

^b^Statistically significant improvement (*P*<.001) across all evaluation.

^c^METEOR: Metric for Evaluation of Translation With Explicit Ordering.

^d^BERTScore: Bidirectional Encoder Representations From Transformers Score.

^e^ROUGE: Recall-Oriented Understudy for Gisting Evaluation.

^f^ROUGE-L: Recall-Oriented Understudy for Gisting Evaluation—Longest Common Subsequence.

^g^BLEU: Bilingual Evaluation Understudy.

### Performance by Metric Category

We analyzed performance across 3 metric categories: lexical overlap, semantic similarity, and embedding-based similarity.

#### Lexical Overlap (BLEU and ROUGE)

Surface-level metrics showed dramatic differences between the 2 models. The generic model achieved very low scores on higher-order n-gram metrics, with BLEU-4 at only 0.81% compared to 21.24% for the tailored model. However, for 4-word phrases (BLEU-4), the generic model dropped to only 0.81%, while the tailored model maintained 21.24%. This shows that the generic model struggles to generate correct long phrases (BLEU-4), while the tailored model performs much better.

#### Semantic Similarity (METEOR)

The detailed model on METEOR, which accounts for stemmed variants, achieved 18.47% versus 15.33% for the generic model. It indicates that detailed instructions enhance linguistic matches.

#### Embedding-Based Similarity (BERTScore and SentenceTransformer)

Our analysis showed clear differences between the 2 instruction strategies. The generic model achieved 78.92%, while the detailed model reached 87.05%. The high score of 87.05% achieved by the detailed model suggests that its outputs are clinically reliable and closely align with how physicians typically document discharge instructions. This finding is strongly confirmed by SentenceTransformer results, where the detailed model achieved 74.90% compared to only 11.91% for the generic model. Together, these metrics indicate that detailed instructions produce clinically appropriate discharge instructions.

Cross-model validations revealed that phrase-level (BLEU-4) and bigram overlap (ROUGE-2) improved for all models. However, ROUGE-L gains were modest but consistent. BERTScore changes were very small. Detailed instructions improved phrase-level accuracy and structural alignment consistently, while semantic similarity stayed stable (Table 5).

**Table 5:** Cross-model validation (n=200 per model) comparing basic and detailed prompting using GPT-4, GPT-3.5-turbo, Mistral-7B, Qwen2.5-3B, and Llama-3.2-3B.

Model and prompt			BLEU-4^a^	ROUGE-L^b^		BERTScore^c^
**GPT-4 [19]**						
	Basic	0.018		0.131	0.809	
	Detailed	0.045		0.169	0.821	
**GPT-3.5-turbo [20]**						
	Basic	0.023		0.139	0.818	
	Detailed	0.037		0.157	0.819	
**Mistral-7B [21]**						
	Basic	0.024		0.131	0.811	
	Detailed	0.035		0.146	0.811	
**Qwen2.5-3B [22]**						
	Basic	0.014		0.114	0.806	
	Detailed	0.017		0.119	0.807	
**Llama-3.2-3B [23]**						
	Basic	0.019		0.128	0.802	
	Detailed	0.020		0.128	0.803	

^a^BLEU: Bilingual Evaluation Understudy.

^b^ROUGE-L: Recall-Oriented Understudy for Gisting Evaluation—Longest Common Subsequence.

^c^BERTScore: Bidirectional Encoder Representations From Transformers Score.

### Subgroup Performance by Service Category

We performed subgroup analysis across 6 clinical service categories representing 3621 discharge instructions: general medicine, general surgery, specialized surgery, neurosciences, obstetrics and gynecology, and psychiatry or emergency. Table 6 presents the performance improvements for each service category.

All service categories showed BERTScore gains with the tailored model, particularly surgical departments, where general surgery rose by +0.140 and specialized surgery by +0.152 (Table 6). Three categories showed that BLEU-1 declines, which we interpret as the model demonstrating true language understanding through paraphrasing rather than memorization. Without exception, all subgroups demonstrated enhanced semantic accuracy. These findings confirm that the detailed instruction approach generalizes well. It is not specialty-specific but rather improves documentation across the clinical spectrum.

**Table 6:** Performance improvements by clinical service category.

Service category	BLEU-1^a^ (tailored-generic)	BLEU-4 (tailored-generic)	ROUGE-L^b^ (tailored-generic)	BERTScore^c^ (tailored-generic)
General medicine	–0.021	+0.003	+0.014	+0.075
General surgery	+0.024	+0.003	+0.086	+0.140
Specialized surgery	+0.015	+0.002	+0.072	+0.152
Neurosciences	–0.003	+0.008	+0.052	+0.111
OB/GYN^d^	+0.018	+0.004	+0.073	+0.116
Psychiatry or emergency	–0.027	+0.002	–0.002	+0.051

^a^BLEU: Bilingual Evaluation Understudy.

^b^ROUGE-L: Recall-Oriented Understudy for Gisting Evaluation—Longest Common Subsequence.

^c^BERTScore: Bidirectional Encoder Representations From Transformers Score.

^d^OB/GYN: obstetrics and gynecology.

### Statistical Interpretation

#### Overview

The Wilcoxon signed rank test [24] was used to compare the performance of the generic and tailored models across key evaluation metrics. Results showed statistically significant improvements in the tailored model for all tested metrics (*P*<.001), with consistently large effect sizes as measured by the rank-biserial correlation. Table 7 displays the detailed results for all metrics.

**Table 7:** Statistical comparison of generic and tailored model performance on discharge instruction generation (n=3621).

Metric	*P* value	RBC^a^ (effect size)
BLEU-1^b^	<.001	0.976
BLEU-2	<.001	0.999
BLEU-3	<.001	0.999
BLEU-4	<.001	0.999
ROUGE-1-F^c^	<.001	0.995
ROUGE-2-F	<.001	0.999
ROUGE-L-F	<.001	0.999
METEOR^d^	<.001	0.998
BERTScore^e^-*F*_1_	<.001	0.881
BERTScore-Official	<.001	0.999

^a^RBC: rank-biserial correlation.

^b^BLEU: Bilingual Evaluation Understudy.

^c^ROUGE: Recall-Oriented Understudy for Gisting Evaluation.

^d^METEOR: Metric for Evaluation of Translation With Explicit Ordering.

^e^BERTScore: Bidirectional Encoder Representations From Transformers Score.

#### Results Interpretation

The interpretation of results is as follows:

- BLEU-4 scores, which measure 4-word phrase accuracy: The tailored model achieved a median score of 10.85% (IQR 0.07-0.27) compared to 0.39% (IQR 0.00-0.01) for the generic model (*P*<.001; *r*=0.9997), indicating a substantial improvement in generating accurate longer medical phrases.
- ROUGE-L scores: The tailored model had a median score of 23.73% (IQR 0.19-0.49) versus 8.69% (IQR 0.06-0.11) for the generic model (>*P*<.001; *r*=0.9990), reflecting better structural alignment with reference instructions.
- BERTScore scores: The tailored model reached a median similarity of 92.17% 0.86 (IQR 0.85-0.89) compared to 84.10% (IQR 76.0-81.0) for the generic model (>*P*<.001; *r*=0.8807), confirming improved semantic faithfulness.

The large effect sizes indicate that the observed improvements are not only statistically significant but also practically meaningful in real-world clinical contexts.

#### Distribution Analysis of Model Performance

**Figure 4:**
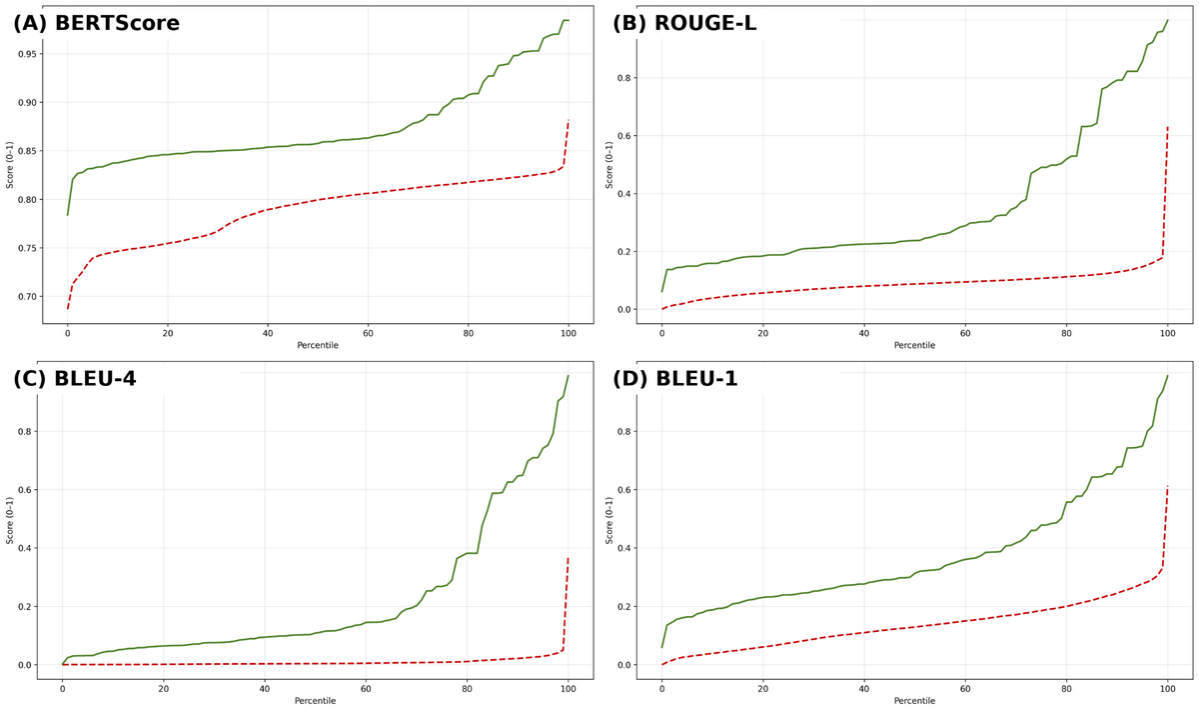
Percentile distributions of test-set scores (n=3621): (A) BERTScore, (B) ROUGE-L, (C) BLEU-4, and (D) BLEU-1. The detailed model (solid line) consistently exceeds the generic model (dashed line) across percentiles. BERTScore: Bidirectional Encoder Representations From Transformers Score; BLEU: Bilingual Evaluation Understudy; ROUGE-L: Recall-Oriented Understudy for Gisting Evaluation—Longest Common Subsequence.

We examined score distributions across all 3621 test samples. Table 8 reports percentile values (0%, 25%, 50%, 75%, 100%) for the 4 metrics: BLEU-1 (word overlap), BLEU-4 (phrase overlap), ROUGE-L-F (structural alignment), and BERTScore-Official (semantic similarity). Figure 4 visualizes the full percentile curves across metrics performance.

**Table 8:** Percentile distribution of BLEU-1^a^, BLEU-4, ROUGE-L^b^, and BERTScore^c^ metrics for generic and tailored models (n=3621).

Metric and model			Range		Median (IQR)
**BLEU-1**					
	Generic	0.0-0.61		0.13 (0.07-0.19)	
	Tailored	0.06-0.99		0.31 (0.24-0.48)	
**BLEU-4**					
	Generic	0.0-0.37		0.0 (0.0-0.01)	
	Tailored	0.0-0.99		0.11 (0.07-0.27)	
**ROUGE-L**					
	Generic	0.0-0.63		0.09 (0.06-0.11)	
	Tailored	0.06-1.0		0.24 (0.19-0.49)	
**BERTScore**					
	Generic	0.69-0.88		0.81 (0.76-0.81)	
	Tailored	0.78-0.98		0.86 (0.85-0.89)	

^a^BLEU: Bilingual Evaluation Understudy.

^b^ROUGE-L: Recall-Oriented Understudy for Gisting Evaluation—Longest Common Subsequence.

^c^BERTScore: Bidirectional Encoder Representations From Transformers Score.

#### Clinical Relevance

From a medical standpoint, the improvements observed with the tailored model are substantial:

- BLEU-4 captures the model’s ability to reproduce clinically relevant multiword expressions. The higher scores suggest that the tailored model better replicates the language typically used by health care providers in discharge documentation.
- ROUGE-L, which reflects the overall structural similarity to the reference text, indicates that the tailored model produces more coherent and well-organized discharge instructions, crucial for patient comprehension and safety.
- BERTScore, a semantic similarity measure, shows that the tailored model generates outputs that are more meaningfully aligned with physician-written references, reducing the risk of ambiguity or misinformation.

These results suggest that fine-tuning with detailed, medically grounded instructions tailored to the hospital setting produces discharge instructions that are more accurate and clinically appropriate. This supports improved communication between care teams and patients. For a comparative example of model versus physician-written discharge instructions, see Multimedia Appendix 3. Illustrative best, worst, and typical cases, aligned with physician references and metric outputs, are also included.

### Qualitative Error Analysis

We reviewed representative cases, in which the basic and detailed models demonstrated omission and hallucination errors. To illustrate the safety implications of these errors, we first present a medication-omission case (Textboxes 1-3).

**Case 1: comparison of basic and detailed model outputs showing improved capture of medication adjustments.**

In the reference written by the physician, multiple medication changes were documented, including warfarin, Lovenox, metoprolol, atenolol, amlodipine, and hydrochlorothiazide. The following excerpt shows these critical modifications: “We made the following changes to your medications: • lowered the dose of the blood pressure medication Amlodipine • stop taking Hydrochlorothiazide (HCTZ) • stop taking Atenolol 100 mg daily (we started Metoprolol instead) • take Warfarin (the blood thinner) as directed • use Lovenox injections until Warfarin is at a therapeutic level ...”

The basic model’s output contained no medication instructions, omitting all medication-related information entirely. Although the detailed model removed the explicit information about discontinuing atenolol, it captured the essential substitution with metoprolol and the critical anticoagulation therapy: “You were started on a new medication called metoprolol… continue your blood thinners called lovenox and warfarin.” We then examined a hallucination case, where the output included clinical features absent from the reference.

**Case 2: example of physician-documented vertigo presentation demonstrating that the detailed model maintained diagnostic accuracy while the generic model introduced hallucinated symptoms.**

In the ground truth, the physician documented a straightforward presentation of vertigo and nausea episodes occurring at the primary care office, with normal examination findings and negative imaging for stroke. The reference explicitly stated: “You were admitted with a few episodes of vertigo and nausea and vomiting while at your PCPs office ... MRI of your brain which did not show any evidence of acute stroke ... This symptom likely represents a peripheral vertigo.”

The generic model fabricated entirely different symptoms. Instead of the vertigo case, it produced: “Chief Complaint: worsening aphasia and hallucination ... left-handed man with non-ischemic cardiomyopathy ...” This output contained multiple false features not found in the original reference, such as aphasia, hallucinations, and a complex cardiac problem. These features represent hallucinations that could misdirect care.

In contrast, the detailed model captured the actual clinical presentation: “You were admitted to the hospital for dizziness ... evaluated for a stroke but we did not find any evidence of this on your brain imaging. We also started you on a medication called valium which helped with your symptoms. You should only take this medication as needed for your dizziness.” While the detailed model simplified the output, it remained aligned with the reference-documented vertigo presentation; however, it hallucinated a new medication (Valium) not present in the reference.

**Case 3: comparison showing the generic model’s incorrect dietary recommendations versus the detailed model’s clinically consistent output.**

This case demonstrates diet guidance errors. In the ground truth, the physician documented a patient with Crohn disease recovering from small bowel obstruction. The key instructions included monitoring bowel function, and the reference noted: “You have tolerated a regular diet, are passing gas and your pain is controlled with pain medications by mouth.”

The generic model generates a special diet: “gradually increase your intake of clear liquids and light foods ... Avoid foods that are high in fat, fiber, or are spicy.” This contradicted the reference, which stated the patient was already “tolerated a regular diet.”

The detailed model generated the clinical condition correctly and warning signs but simply did not address diet at all, which aligns with the reference document that also provided no specific dietary guidance beyond, noting the patient had tolerated a regular diet.

Beyond content errors, we observed that the models’ writing styles often differed significantly from how physicians write discharge instructions. In our dataset, physician-authored instructions consistently used plain text without special formatting or Markdown symbols; accordingly, the generated text should adhere to institutional formatting conventions.

However, the basic model consistently ignored this convention. For example, it used “###” Markdown headers in 100% of outputs, elements that never appeared in the references. The detailed model performed notably better at matching clinical writing patterns. It generated text similar to physician notes and completely avoided the use of “###” Markdown headers (0%).

### Clinical Safety Evaluation by Experts

Overall, the tailored model markedly improved safety compared with the generic model. For the generic model, ratings were safe in 3 of 40 (7.5%) cases, minor safety issues in 3 of 40 (7.5%) cases, and not safe in 34 of 40 (85%) cases. For the tailored model, ratings were safe in 16 of 40 (40%) cases, minor safety issues in 22 of 40 (55%) cases, and not safe in 2 of 40 (5%) cases.

Per-service analysis revealed consistent improvements across all departments (Table 9). Most notably, surgery services showed complete elimination of unsafe instructions with the tailored model compared to the generic model.

**Table 9:** Human evaluation of discharge instruction safety by clinical service and model type.

Service and model			Safe, n (%)		Safe but needs minor changes, n (%)		Not safe at all, n (%)
**Orthopedics**							
	Generic	0 (0)		0 (0)		10 (100)	
	Tailored	3 (7.5)		7 (70)		0 (0)	
**Neurology**							
	Generic	3 (7.5)		0 (0)		7 (70)	
	Tailored	5 (50)		4 (40)		1 (10)	
**Surgery**							
	Generic	0 (0)		0 (0)		10 (100)	
	Tailored	4 (40)		6 (60)		0 (0)	
**Medicine**							
	Generic	0 (0)		3 (7.5)		7 (70)	
	Tailored	4 (40)		5 (50)		1 (10)	
**Totals (n=40)**							
	Generic	3 (7.5)		3 (7.5)		34 (85)	
	Tailored	16 (40)		22 (55)		2 (5)	

Across the 4 clinical services, the tailored model produced discharge instructions ready for clinical use in 16 of 40 (40%) cases, with 22 of 40 (55%) cases requiring only minor edits. Despite this improvement, the 5% (2/40) unsafe rate confirms that physician review remains a critical safety step.

## Discussion

### Principal Findings

Despite using the same underlying base model (Mistral-NeMo-Instruct), the model fine-tuned with detailed, medically tailored instructions substantially outperformed the model fine-tuned with basic, generic instructions across all evaluation metrics. This highlights the critical importance of specific, clinically grounded instruction tuning that is aligned with the practices of the target hospital.

The improvements were observed across all the BLEU scores, where the tailored instruction model consistently outperformed the generic model. The tailored model generates words and phrases that closely match those found in human-written reference texts. It indicated its ability to generate context-appropriate discharge instructions.

The higher ROUGE-L score means that the ordering of words in the generated text closely matches the reference. This helps ensure that the discharge instructions are organized and easier to follow. The final result of ROUGE-L suggests that the tailored model produces discharge instructions with better logical flow, improved readability, and a style that closely follows how doctors write instructions for patients.

BERTScore reflects whether the generated text preserves the meaning of the reference text. For example, it recognizes that “take medicine twice daily” and “take medication 2 times per day” convey the same instruction. The high score (87%) shows that the tailored model captures medical meaning correctly. This semantic accuracy is crucial for patient safety. When the model generates instructions about drug interactions or warning signs, it must convey the correct medical message. Our results show that the tailored model does this reliably. In practical terms, physicians can trust that the generated instructions maintain clinical accuracy while reviewing and finalizing them.

The human evaluation findings provide important validation of our automated metrics while highlighting important safety considerations for clinical deployment. The physician safety assessment revealed that only 40% of tailored model outputs were immediately safe for patient use, with an additional 55% requiring minor modifications. Most concerning is that the 5% rated by physicians as unsafe raises important patient-safety concerns, underscoring the need for clinician oversight and continuous monitoring prior to clinical use.

Although both models used the same training data, the tailored model performed much better. The only difference was the design of the instructions. This shows how we instruct the model is important. The detailed instructions gave the model clear guidance about discharge instructions. It specified what information to include. The generic instructions missed this guidance.

This finding has practical value for health care systems. Each hospital can craft detailed prompts (instructions) that match its specific documentation standards and use these instructions for fine-tuning. The same base model adapts to each setting simply by changing the instructions and fine-tuning the model with the desired detailed instructions. This highlights an important shift in strategy: instead of collecting more training data or using larger models, health care systems can improve artificial intelligence performance through better instruction design. This approach is faster and less expensive than other methods.

These encouraging results prompted us to investigate service-specific performance. We examined whether the benefits of detailed instructions extended across 6 clinical service categories. Improvements were observed in all groups, but the size of the gains varied. Surgical specialties achieved BERTScore gains roughly twice that of general medicine. These gains make clinical sense because surgical discharge instructions mostly follow consistent procedural descriptions and template-like postoperative protocols, contrasting sharply with internal medicine, which needs to address multiple conditions, medication interactions, and individualized care plans.

Additionally, a revealing pattern appeared in general medicine, neurosciences, and psychiatry or emergency, where lower BLEU-1 scores coincided with higher BERTScore performance. This apparent contradiction actually represents a strength: the model learned to express medical concepts accurately without rigid adherence to source text phrasing.

All departments converged to the same performance range. Such consistent results justify implementing this across the entire institution.

### Comparison With Earlier Works

Early approaches to discharge summary generation relied heavily on templates. While this method ensured consistency, it often lacked personalization and failed to capture patient-specific needs. The introduction of neural language models marked a significant shift in solving the problem of personalized patient instructions. An early study on natural language generation for EHRs established that encoder-decoder models could produce realistic emergency chief complaints text. These are short texts that describe why a patient came to the emergency room. This study demonstrated that neural networks could produce realistic text. However, this paper worked with short chief complaints, not full discharge instructions. In addition, the author used a very simple instruction with just 21 words: “You are a doctor. Generate discharge instructions for the following patient based on their discharge summary” [25].

Recent research has focused on improving generated discharge summaries using medical language models. The MEDISCHARGE study [26] fine-tuned Meditron-7B, a medical language model, to write discharge instructions. They made several technical improvements: extending the model’s reading capacity from 2000 to 6000 words and adding methods to extract information from different clinical sections. Their results on MIMIC-IV showed BLEU-4 of 0.103, ROUGE-L of 0.284, and BERTScore of 41.7%. However, their approach needs complex data processing and powerful computers, making it difficult for many hospitals to use [26].

Our work takes a different path by focusing on instruction quality rather than architectural complexity. We show that writing better instructions for the model can achieve improved results. Our instruction fine-tuning achieved BERTScore of 87.05% versus MEDISCHARGE’s 41.7% [26], showing the effectiveness of detailed instructions. We achieved this by giving the model clear instructions, which included specific medical guidelines, defined roles, and example texts. These results show that detailed instruction engineering alone can yield substantial improvements, offering a practical alternative for clinical text generation without requiring system-level modifications.

Another advanced approach is Re3Writer, developed by Liu et al [27]. This approach works in 3 steps. First, it finds similar patient cases. Second, it uses a medical knowledge graph database to understand medical relationships. Third, it combines all this information to write patient instructions. It achieved a BLEU-4 of 30.5 on the transformer model as a backbone. Despite these achievements, their approach requires maintaining a database of historical cases and constructing medical knowledge graphs, which added considerable complexity to the deployment pipeline [27]. A summary comparison of performance across earlier works and our approach is presented in Table 10.

**Table 10:** Performance comparison of discharge instruction generation methods.

Study or approach	Model or method	Dataset	BLEU-4^a^	ROUGE-L^b^	BERTScore^c^ (%)	Key Limitations
Lee (2018) [25]	Encoder-decoder (early NLG^d^ for EHR^e^)	Chief complaints	—^f^	—	—	Worked only with short texts
MEDISCHARGE (Wu et al, 2024) [26]	Fine-tune a medical model (Meditron-7B) with dynamic section-based extraction	MIMIC-IV^g^	0.103	0.284	41.7	Complex preprocessing
Re3Writer (Liu et al, 2024) [27]	Retrieval+medical knowledge graph transformer backbone	MIMIC-III^h^	30.5	42.2	—	Requires maintaining knowledge graphs; deployment complexity
Our work (2025)—generic instructions+fine-tuning	Fine-tuned Mistral-NeMo fine-tuned with simple instructions	MIMIC-IV	0.81	8.59	78.92	Generated text lacked structure
Our work (2025)—detailed instructions+fine-tuning	Fine-tuned Mistral-NeMo fine-tuned with detailed instructions	MIMIC-IV	21.24	26.52	87.05	Generated text required clinician oversight

^a^BLEU: Bilingual Evaluation Understudy.

^b^ROUGE-L: Recall-Oriented Understudy for Gisting Evaluation—Longest Common Subsequence.

^c^BERTScore: Bidirectional Encoder Representations From Transformers Score.

^d^NLG: natural language generation*.*

^e^EHR: electronic health record.

^f^Not available.

^g^MIMIC-IV: Medical Information Mart for Intensive Care IV.

^h^MIMIC-III: Medical Information Mart for Intensive Care III.

### Feasibility of Open-Source Models for Discharge Instruction Generation

The results achieved by our tailored model are particularly promising, demonstrating that high-quality discharge instruction generation is possible using an open-source, fine-tuned language model. Despite not relying on proprietary large-scale systems like GPT-4 [19], our tailored model produced outputs with significantly higher lexical, structural, and semantic accuracy across all metrics. Remarkably, our model achieved a BERTScore of 87.2%, which gives a strong indication of better alignment with doctor-written instructions. These findings underscore the potential of fine-tuned, open-source models to deliver clinically useful results. This makes the approach not only cost-effective and accessible but also compliant with health care–specific data standards. It offered a path for hospitals aiming to integrate generative AI into documentation workflows without compromising control, privacy, or quality.

### Cross-Model Validation: Small-Model Benefits in Resource-Limited Settings

To explore whether smaller language models could benefit from our instruction design, we conducted a preliminary evaluation across multiple models using 200 discharge instructions. The preliminary results suggest promising possibilities for resource-constrained settings. In 200 cases, GPT-4 [19] showed the largest improvements (150% for BLEU-4), revealing that it has advanced instruction-following capabilities. Mistral-7B [21] reached ROUGE-L 0.146, close to GPT-3.5-turbo [20] at 0.157, while using much less compute. Qwen2.5-3B [22] improved BLEU-4 by 20.4% and ROUGE-2 by 20.8%. These results suggest the possibility that careful instruction design helps small models produce more accurate medical phrases and term pairs. Although these initial findings on 200 samples cannot confirm broad generalizability, they indicate that our approach of detailed instructions with compact models may have a promising direction to be deployed in resource-limited settings.

These findings are consistent with the broader understanding that larger instruction-tuned models tend to capture complex instructions more effectively than their smaller counterparts, while smaller models can still benefit significantly from optimized instruction formatting [9].

### Workflow Integration and Time Efficiency

During our inference, the tailored model generated discharge instructions for a single patient case within 30-40 seconds. This represents a promising direction for assisting physicians with documentation tasks. In contrast, current manual discharge processes typically require 15-30 minutes of physician time per patient [6].

These inference times (30-40 seconds per case) are dependent on several technical factors, including network latency and the computational hardware used in our experimental setup. Importantly, our model examined cases one at a time in a controlled experimental environment. However, the total time savings in practice will depend on multiple factors beyond generation speed, including the hospital’s available hardware resources, the time required for physicians to review and modify the drafted generated text, the frequency of cases requiring substantial versus minor edits during revision, and system performance under real-world conditions with multiple simultaneous users.

This efficiency gain may ease documentation burdens, providing particular value in high-volume discharge settings. However, it is important that implementation efforts emphasize scalable system design and include comprehensive evaluations to assess real-world time savings and physician satisfaction.

### The Critical Role of Clinician Oversight

Our evaluation revealed a 5% unsafe rate, demonstrating that oversight frameworks are not optional but essential in clinical deployment. This result illustrates that even with advanced LLMs, certain cases, particularly those involving complex patient needs or specific clinical reasoning, require close expert review. These medical cases provide a clear reminder that human judgment cannot be replaced and that LLM should be positioned as a supportive tool.

This finding affirms the value of human-LLM collaboration in drafting patient discharge instructions. The LLM accelerates the drafting process, while physicians refine the outputs by applying their clinical expertise. Such collaboration mitigates risk and transforms the LLM into an opportunity to save physicians’ time. In addition, routine physician reviews create a feedback cycle that fosters both the model’s strengths and areas for improvement. These reviews can enhance future model refinements and provide a guide for the model’s optimization.

The persistent problem of LLM hallucinations further reinforces the necessity of human oversight. Without physicians’ review, there is a chance to generate discharge instructions that misrepresent patient care plans, potentially compromising safety. At the current time, success depends on developing interaction models in which LLMs contribute efficiency, while physicians ensure safety and accuracy in the care plan.

### Limitations

Our study was conducted under significant resource constraints, which limited the scope of experimentation. We were only able to fine-tune and evaluate a single open-source language model due to limited computing time and hardware access. As a result, the effects of instruction variation were not tested across different model sizes or architectures.

The inference time measurements were obtained on limited hardware resources during the experiment, where the model processed cases sequentially, one at a time. We did not conduct stress testing to evaluate performance under multiple concurrent cases, as would occur in a busy hospital with several physicians generating discharge instructions simultaneously. Furthermore, inference times in our setup depend on network and hardware conditions and may not generalize to clinical environments.

Our human evaluation was limited to 40 discharge summaries across 4 specialties. It was not large enough to capture the full variability of clinical practice. While this sample provided valuable potential insights, future studies should expand human evaluation with a larger sample size and more diverse specialties and calculate the formal interrater agreement metrics to better establish the reliability of clinical safety assessments.

Our evaluation is physician-centered, focusing on the goal of helping clinicians reduce their documentation burden. Future studies should therefore integrate patient-centered assessments. Such evaluations can capture aspects beyond clinical safety, including readability, freedom from criticism, avoidance of bias, encouragement to follow instructions, a welcoming tone that motivates patients to seek medical advice when needed, and clarity in presenting guidance on medications, activities, warning signs, and when to seek further help.

### Conclusions

This study demonstrated that fine-tuning open-source LLMs with detailed, tailored instructions significantly enhanced the quality of generated discharge instructions compared to generic prompts. The statistical analysis across multiple evaluation metrics revealed substantial and practically meaningful improvements. The tailored, detailed model generated outputs closely aligned with real instructions written by doctors. These findings suggested the importance of customizable instructions in leveraging open-source language models in hospitals. The tailored instruction tuning method offered a promising, cost-effective, efficient, and scalable strategy to automate discharge instruction generation, thereby potentially reducing clinician workload and improving patient safety and comprehension in hospital care settings.

## Appendix

Multimedia Appendix 1 Complete text of the detailed instruction prompt (587 words) used for model fine-tuning.

Multimedia Appendix 2 Discharge instruction safety rating rubric.

Multimedia Appendix 3 Example discharge instructions comparing physician-written reference text with generic and tailored model outputs across best, median, and worst-case scenarios.

## Abbreviations

BLEU: Bilingual Evaluation Understudy
BERTScore: Bidirectional Encoder Representations From Transformers Score
EHR: electronic health record
GPU: graphics processing unit
HIPAA: Health Insurance Portability and Accountability Act
LLM: large language model
METEOR: Metric for Evaluation of Translation With Explicit Ordering
MIMIC-IV: Medical Information Mart for Intensive Care IV
ROUGE: Recall-Oriented Understudy for Gisting Evaluation
ROUGE-L: Recall-Oriented Understudy for Gisting Evaluation—Longest Common Subsequence

## Data Availability

The data used in this study were derived from the publicly available Medical Information Mart for Intensive Care IV database, which is hosted on PhysioNet [12]. Access to Medical Information Mart for Intensive Care IV requires completion of the Collaborative Institutional Training Initiative “Data or Specimens Only Research” course and acceptance of the PhysioNet Credentialed Health Data Use Agreement. Due to data use agreement restrictions and patient privacy considerations, the raw data cannot be shared directly. However, the datasets generated and analyzed during this study are reproducible using the described cohort extraction criteria. The code for model fine-tuning and evaluation, along with implementation scripts for both the generic and detailed instruction-tuning approaches, is openly available on Zenodo [28].
