# Supplementary material for: Impact of Detailed Versus Generic Instructions on Fine-Tuned Language Models for Patient Discharge Instructions Generation: Comparative Statistical Analysis
Source: JMIR Form Res. 2025 Oct 30;9:e80917. doi: 10.2196/80917 (PMC12616184; doi:10.2196/80917)
Supplement: Multimedia Appendix 1 [file formative_v9i1e80917_app1.docx]

## A. Complete Text of Detailed Prompt

Below is the complete text of the detailed prompt (587 words) used in the manuscript titled "Impact of Detailed Versus Generic Instructions on Fine-Tuned Language Models for Patient Discharge Instructions Generation: Comparative Statistical Analysis.

| **1. Think and Analyze Before Generating Instructions:**   - Carefully read and understand the context of the entire discharge summary before extracting information. - Identify and extract the key medical details, particularly medications, treatments received, admission reasons, warning signs, and follow-up care. - Ensure that no essential information is omitted, and cross-check for potential errors. - If a key piece of information isn’t present in the summary, omit it. - Extract information directly from the discharge summary without adding or fabricating details. - Reduce hallucinations by verifying each fact against the original discharge summary.   **2. Cover All Relevant Medical Information to ensure Completeness:**  1. **Understand the Case Context**   - Analyze the discharge summary to extract the primary diagnosis, secondary conditions, and the overall context of the patient's hospital stay, including the purpose of hospitalization, key findings, and procedures or therapies received.   2. **Admission Reason**   - State the primary reason for hospitalization. - Include only relevant diagnoses that impact discharge care if explicitly stated.   3. **Treatment Received**   - Summarize key procedures, interventions, or treatments. - Focus on treatments requiring post-discharge management. - Do not elaborate beyond what’s stated in the discharge summary.   4. **Medications and Usage** List prescribed medications with details on:   - Names, dosages, and frequencies. - Administration instructions (e.g., before/after meals). - Duration of use, if specified. - Do not infer or assume medication instructions not explicitly stated. - Use exact medication names and dosages from the discharge summary.   5. **Follow-Up Care** Include follow-up actions such as:   - Scheduling follow-up appointments. - Specific tests or procedures to monitor recovery. - Referrals to specialists or home care providers.   6. **Warnings and Red Flags**   - Clearly list specific symptoms requiring medical attention. - Indicate the appropriate action for each warning sign (e.g., call doctor vs. go to emergency department). - Only include warning signs explicitly mentioned in the source document.   **3. Ensure Clarity and Structure to Readability:**  Maintain a **logical flow** with this preferred sequence:   1. Introduction (e.g., "It was a pleasure taking care of you at the hospital.") 2. Admission reason 3. Key treatments received 4. Medications with detailed instructions 5. Activity & lifestyle recommendations (if present) 6. Follow-up care requirements 7. Warning signs & when to seek help 8. Closing reassurance   **4. Tone: Patient-Friendly and Reassuring:**   - Use positive and patient-friendly language. - Avoid medical jargon; if necessary, provide layman’s explanations. - Address the patient directly (e.g., "You should take your medication as prescribed"). - Encourage adherence to instructions gently (e.g., "It’s important that you..." or "Please remember to..."). - Ensure the instructions are understandable and patient focused. - Mimic the tone and style of original discharge instructions. - Preserve common expressions found in source documents.   **5. Verify for Patient Safety:**   - The quality of these discharge instructions will impact patient health and safety. - Missing or incorrect details about medications, activities, and warning signs could cause immediate patient harm or trigger complications. - Verify every detail thoroughly to ensure complete and accurate information is provided   **6. Mimicking Real Discharge:**   - Study and replicate the style of actual discharge instructions.   **7. Keep original information:**   - Use exact n-grams from the source document whenever possible. - Prioritize word-for-word extraction over summary or paraphrase. |
| --- |

## B. Complete Text of Generic Prompt

You are a doctor. Generate discharge instructions for the following patient based on their discharge summary.
