# Supplementary material for: Impact of Detailed Versus Generic Instructions on Fine-Tuned Language Models for Patient Discharge Instructions Generation: Comparative Statistical Analysis
Source: JMIR Form Res. 2025 Oct 30;9:e80917. doi: 10.2196/80917 (PMC12616184; doi:10.2196/80917)
Supplement: Multimedia Appendix 3 [file formative_v9i1e80917_app3.docx]

This appendix provides examples of discharge instructions comparing Ground Truth (doctor-written), Generic Model outputs, and Tailored Model outputs. We illustrate best case, general (median), and worst case scenarios from the Generic model side, with corresponding Tailored model outputs.

## Best Case (Highest Generic BERTScore = 0.88)

### Ground Truth (Doctor):

Microdiscectomy

You have undergone the following procedure: microdiscectomy.
Please avoid heavy lifting. Follow up in clinic in 2 weeks.
Contact your doctor if you develop fever, severe pain, or neurological changes.

### Generic Model Output (BERTScore = 0.882):

### **Please follow these instructions:**

1. You have undergone a microdiscectomy procedure.
2. Avoid heavy lifting for 2 weeks.
3. Follow up with your doctor in 2 weeks.
4. Return if you have fever, worsening pain, or weakness.

### Tailored Model Output (BERTScore = 0.903):

You were admitted to the surgery service at ___ hospital and underwent a microdiscectomy.
Avoid heavy lifting. Follow up in clinic in 2 weeks.
Return if fever, severe pain, or neurologic symptoms occur.

## General / Median Case (Generic BERTScore ≈ 0.80)

### Ground Truth (Doctor):

Dear Ms. ___,

It was a pleasure taking care of you during your admission.
Please continue all your home medications.
Follow up with your primary care doctor in 1 week.
Return to the ER for chest pain, shortness of breath, or worsening symptoms.

### Generic Model Output (BERTScore = 0.799):

### **Please see instructions below:**

Please continue your home medications as prescribed.
Follow up with your doctor in 1 week.
Return to the ER if chest pain, shortness of breath, or worsening symptoms.

### Tailored Model Output (BERTScore = 0.833):

Dear Ms. ___,

It was a pleasure taking care of you in the hospital.
Please continue your home medications.
Follow up with your primary care physician in 1 week.
Return to the ER for chest pain, shortness of breath, or worsening symptoms.

## Worst Case (Lowest Generic BERTScore = 0.69)

### Ground Truth (Doctor):

Mr. ___,

You were admitted for fever and chills.
Continue your antibiotics as prescribed.
Drink plenty of fluids.
Follow up with your doctor in 1 week.
Return to the ER if fever, confusion, or worsening symptoms.

### Generic Model Output (BERTScore = 0.687):

### **Please see patient discharge instructions:**

Admission: fever and chills.
Medications: continue.
Follow up with physician.
(The output is very terse, missing antibiotic details and emergency guidance.)

### Tailored Model Output (BERTScore = 0.842):

You were admitted to the hospital because of fever and chills.
Continue your antibiotics as prescribed.
Drink plenty of fluids.
Follow up with your doctor in 1 week.
Return to the ER if fever, confusion, or worsening symptoms.
